# A simple mechanistic terminology of psychoactive drugs: a proposal

**DOI:** 10.1007/s00210-020-01918-x

**Published:** 2020-06-13

**Authors:** Roland Seifert, Bastian Schirmer

**Affiliations:** Institute of Pharmacology, Hannover Medical School, Carl-Neuberg-Str. 1, 30625 Hannover, Germany

**Keywords:** Psychoactive drugs, Drug nomenclature, Pharmacology

## Abstract

Antidepressants, antiepileptics, mood stabilizers, and antipsychotics are extremely broadly used psychoactive drugs. These drug terms are universally used in the literature. However, the indications of these drugs have broadened substantially and overlap. The mismatch between drug classification and clinical uses causes a lot of confusion in communication and renders literature searches increasingly difficult. Therefore, we propose to drop the above terms altogether and replace them by simple mechanistic terms. Antidepressants are re-named as norepinephrine/serotonin (NE/5-HT) enhancers, antiepileptics comprising drugs with different mechanisms become neuronal inhibitors with pleiotropic effects (NIPEs), and antipsychotics become antagonists at multiple G protein–coupled receptors (mGPCR antagonists). Alkali metal ions, comprising lithium, are integrated into NIPEs. The terms “typical/first-generation/conventional” and “atypical/second-generation/non-conventional” antipsychotics should be dropped, because the original criterion for distinction, i.e., the presence and absence of extrapyramidal motor effects, respectively, is not valid anymore. The suggested changes in drug nomenclature have already been implemented into a recent textbook (Seifert R, *Basic Knowledge of Pharmacology*). The revised nomenclature ensures consistency with other fields of pharmacology and assignment of drug classes to indications without causing confusion. The authors acknowledge that the change in drug nomenclature is a cultural process that will take time and openly discuss the problems associated with the proposal. Ultimately, international learned societies will have to agree on a new nomenclature.

**Electronic supplementary material:**

The online version of this article (10.1007/s00210-020-01918-x) contains supplementary material, which is available to authorized users.

## The status quo of psychoactive drug terminology

**Fig. 1:**
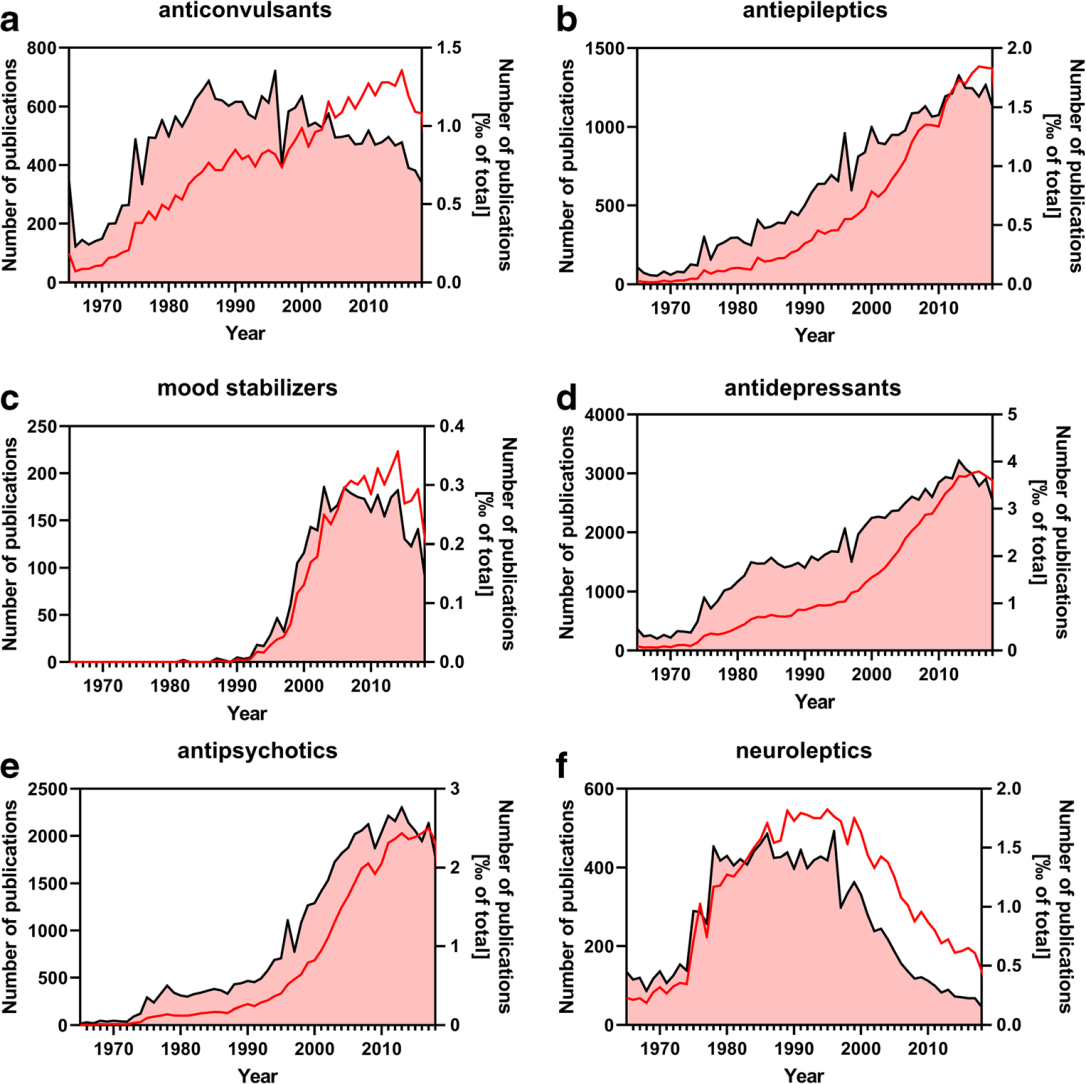
Frequency of the use of several broad terms for psychoactive drugs in the medical literature (1965–2018). The PubMed search was performed on 08 May 2020 and was confined to titles and abstracts of searchable items. Only publications of the type “journal article” that were referenced in the MEDLINE database were accepted as hits. Depicted in the plots are the absolute number of publications per year that use a specific pharmacological term (red line/left y-axis) and the relative number of these publications normalized to the total number of MEDLINE-referenced publications of the corresponding year (black line with red filling/right y-axis). The exact search conditions are stated in Supplementary Table 1. Please note that the scale of the y-axes is different among the panels

Psychoactive drugs are extremely broadly prescribed both by psychiatrists and general practitioners. Traditionally, psychotropic drugs are divided into *antidepressants*, *antiepileptics* (or anticonvulsants), *mood stabilizers*, and *antipsychotics* (originally designated as neuroleptics). These terms are based on the traditional indications for these drugs. In the literature, the terms are broadly used: Fig. 1 shows the frequency of use of these terms in the medical literature based on a recent PubMed search. Both absolute numbers and relative numbers of the use of terms are reported. The legend of Fig. 1 describes the general search strategy and Supplementary Table 1 describes the specific search terms used. Supplementary Table 2 shows the source data for the graphs shown in Fig. 1. For the sake of focus of this article, we analyzed only selected classes of psychoactive drugs and did not consider, e.g., barbiturates, benzodiazepines, μ-opioid receptor agonists, and acetylcholine esterase inhibitors.

Originally, antidepressants had been exclusively used for depression, antiepileptics for epilepsy, mood stabilizers for bipolar disorder, and antipsychotics for schizophrenia and acute mania. However, this traditional classification started to erode already many years ago when it became clear that the mood stabilizer lithium can be used as augmentation therapy for depression and that antiepileptics can be effectively used for mood stabilization in bipolar disorder (Kaufman 2011; Nelson et al. 2014; Baldessarini et al. 2019). Paradoxically, while indications had started to expand a long time ago, drug nomenclature remained the same, largely due to language used in pharmacology textbooks.

For *antipsychotics*, the situation is even more complicated. Major adverse reactions of antipsychotics such as haloperidol, particularly in high doses prescribed previously, are extrapyramidal motor symptoms (EPSs). These EPSs, specifically Parkinsonian symptoms, akathisia and tardive dyskinesia, can limit therapy with these drugs (Casey 2006; Divac et al. 2014; Sykes et al. 2017; Hirano 2018). The serious EPSs of the original antipsychotics constituted the motivation to develop antipsychotics with fewer or even no EPSs. Because EPSs are so serious, clinicians and scientists alike aimed at classifying drugs into antipsychotics with EPS risk and antipsychotics without EPS risk.

**Fig. 2:**
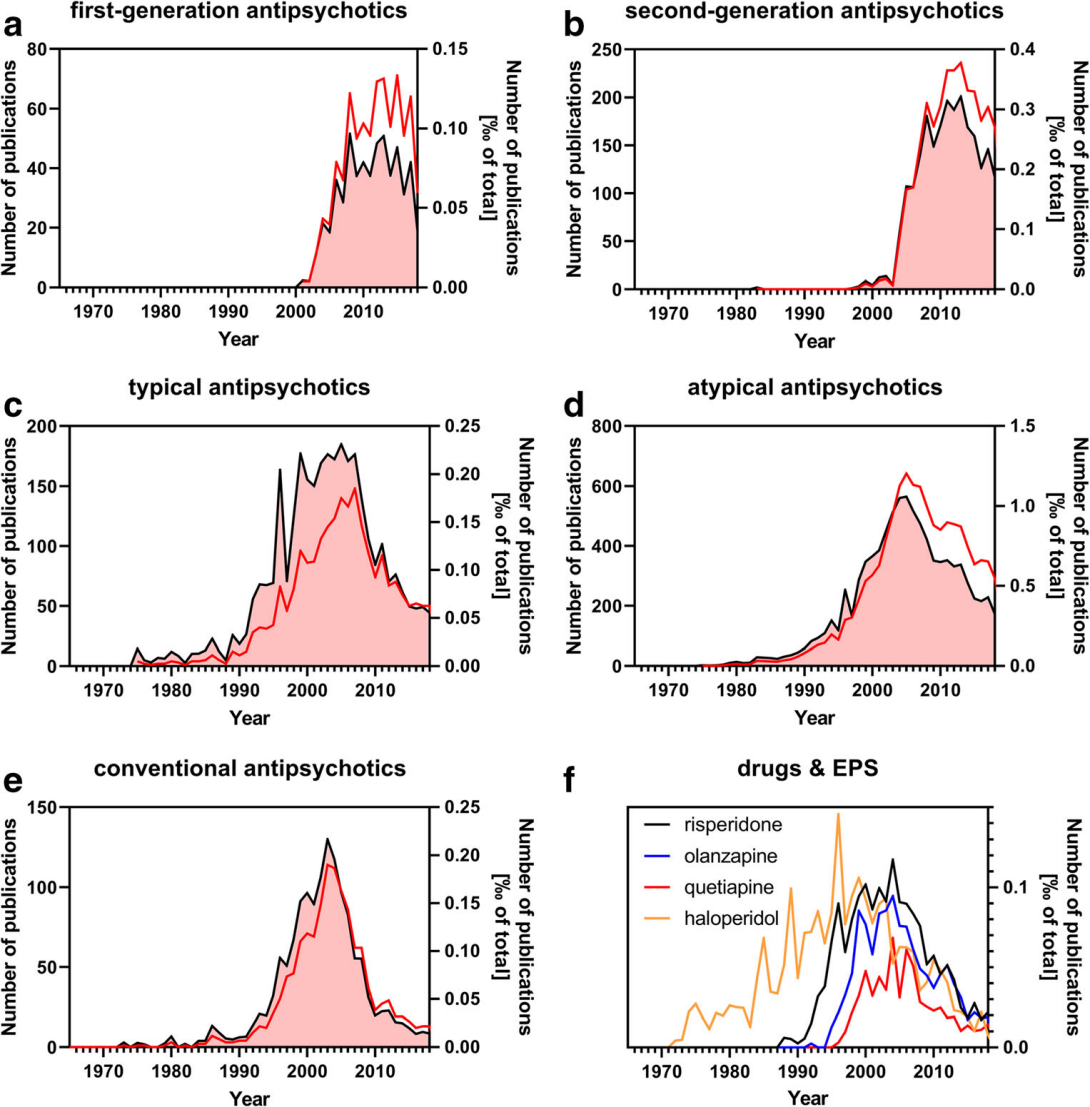
Frequency of the use of terms designating various types of “antipsychotics” in the medical literature (1965–2018). The PubMed search was performed on 08 May 2020 and was confined to titles and abstracts of searchable items. Only publications of the type “journal article” that were referenced in the MEDLINE database were accepted as hits. Depicted in the plots are the absolute number of publications per year that use a specific pharmacological term (red line/left y-axis) and the relative number of these publications normalized to the total number of MEDLINE-referenced publications of the corresponding year (black line with red filling/right y-axis). The exact search terms are stated in the supplement. In panel **f**, the co-occurrence of extrapyramidal side effects (EPS) with each of four exemplary “antipsychotic” drugs in the literature was further analyzed. Please note that the scale of the y-axes is different among the panels. For the sake of clarity, only the relative numbers of publications are shown in panel **f**

Therefore, the terms first-generation and second-generation antipsychotics were coined. Figure 2 shows the absolute and relative frequency of use of these terms in the medical literature based on a PubMed search. The legend of Fig. 2 describes the general search strategy and Supplementary Table 1 describes the specific search terms used. Supplementary Table 3 shows the source data for the graphs shown in Fig. 2. Alternatively to first-generation and second-generation antipsychotics, the terms typical and atypical antipsychotics were introduced. As a third option, the term conventional antipsychotics was introduced, but the term non-conventional or unconventional antipsychotics is not used in the literature. The situation is further complicated by the fact that, instead of the term antipsychotics, the term neuroleptics is used, although now much less commonly than in the past. As a result, we have at least three different terms with the same meaning for a given class of psychoactive drugs, and the terms are arbitrarily used in the literature.

## Use of terms for psychoactive drugs in the literature

*Anticonvulsants* and *antiepileptics* designate one and the same drug class originally developed to treat epilepsies. The term anticonvulsant was initially used more often in absolute numbers than the term antiepileptic, but these days, the term antiepileptic is used more often (Fig. 1a, b). With respect to relation to the disease epilepsy, the term antiepileptics is more appropriate.

The use of the term mood stabilizer has strongly increased since the 1990s and plateaued in recent years (Fig. 1c). The term antidepressant has increased linearly in terms of absolute publications and almost exponentially in terms of relative publications for many years with a plateau during the preceding years (Fig. 1d). The term antipsychotics has increased exponentially in absolute and relative terms for many years and has plateaued recently (Fig. 1e). Until the year 2000, the term antipsychotic competed strongly with the term neuroleptic in the literature (Fig. 1f). However, since the term antipsychotic describes the clinical use of the drugs more precisely (treatment of psychoses) than the term neuroleptic, use of the latter term has declined since the mid-1990s.

The term typical antipsychotics (Fig. 2c) appeared before the term conventional antipsychotics and then followed by first-generation antipsychotics (Fig. 2a, e). The three terms describe the same group of drugs. During recent years, use of the term conventional antipsychotics has declined as has, to a lesser extent, the term typical antipsychotic. Interestingly, the terms non-conventional or unconventional antipsychotics are not used in the literature. The term atypical antipsychotics is still used more commonly than the term second-generation antipsychotics (Fig. 2b, d).

## Problems associated with inconsistent drug nomenclature

There are numerous problems associated with inconsistent drug nomenclature. First of all, the literature search becomes exceedingly difficult and prone to errors and omissions due to the existence of multiple terms for one and the same group of drugs. Not every clinician or researcher may be aware of these duplications or it may be wrongly assumed that different terms have actually a different meaning.

Most importantly, the clinical use of drugs has expanded dramatically over the years. For example, antidepressants are often used for obsessive-compulsive disorder, anxiety disorder, and post-traumatic stress disorder (Skapinakis et al. 2016; Locher et al. 2017; Vries et al. 2018; Amerio et al. 2019; Slee et al. 2019; Stone 2019). Antiepileptics are used for many other diseases than epilepsy including prevention of migraine and trigeminal neuralgia, polyneuropathies, restless leg syndrome, fibromyalgia, and bipolar disorder (Zilliox and Russell 2011; Kaufman 2011; Qin et al. 2018; Hirakata et al. 2018; Salminen and Winkelmann 2018; Parikh and Silberstein 2019), just to mention some indications. Similarly, antipsychotics are used for many more diseases than schizophrenia and acute mania including depression, borderline personality disorders, anxiety disorder, Tourette syndrome, and autism (Ceskova and Silhan 2018; Carulla-Roig et al. 2018; Slee et al. 2019; Baldessarini et al. 2019; Stone 2019; Fallah et al. 2019). The mood stabilizer lithium is used not only for bipolar disorder but also for augmentation in severe depression (Kleeblatt et al. 2017; Baldessarini et al. 2019). Moreover, the use of lithium in neurodegenerative diseases is being explored (Huraskin and Horn 2019; Hampel et al. 2019). These data clearly document an increasing mismatch between drug terminology and clinical drug use. This is also a substantial problem in practical drug prescription and administration. For example, it takes lengthy explanation, why a patient should take an antipsychotic for an anxiety disorder although the patient does not suffer from psychosis. This mismatch may have negative consequences for drug adherence. In addition, the patient may assume that she/he is already so ill that she/he suffers from psychosis and consequently feels worse.

The issue of mismatch between terminology and clinical effects is particularly striking for the atypical and atypical antipsychotics (and synonymous terms). Clinical studies have clearly revealed that the original claim to distinguish between the drug classes, i.e., the presence and absence, respectively, of EPSs, is not valid. Rather, commonly used atypical antipsychotic drugs such as risperidone, olanzapine, and quetiapine possess a significant risk for EPSs (Fischer-Barnicol et al. 2008; Sykes et al. 2017). The only difference between these drugs and haloperidol is that reports on EPSs for haloperidol appeared earlier in the literature (Fig. 2f). This difference is just the result of the fact that risperidone, olanzapine, and quetiapine were introduced later than haloperidol.

## Proposed solution to the problem—Step 1: Get rid of traditional terms

The nomenclature issue has been recognized for some time, and within the discipline of neuropharmacology, a solution has been attempted by developing a multidimensional classification of psychoactive drugs (Zohar et al. 2014, 2015; Rao and Andrade 2016; Caraci et al. 2017). However, it turned out that the nomenclature is too complex, specifically for use in the clinic. Another important issue is that neuropsychopharmacology constitutes only one subfield of pharmacology, and any given nomenclature should be consistent with the nomenclature used in other subfields of pharmacology.

In a recent review, one of the authors of this article proposed to implement, throughout the entire field of pharmacology, a mechanism-based nomenclature of drugs that refrains from using specific indications (Seifert 2018). In fact, the mechanism of action of a drug does not change, but the clinical uses do. In the previous review, the field of neuropsychopharmacology was intentionally not covered because, at that time, a practical solution had not yet been developed.

In the meantime, the process has advanced, and a simple and practical solution can be presented. The proposed nomenclature is simple, uses just few terms, focuses on mechanisms of action, is neutral with respect to clinical uses, and is consistent with nomenclature in other fields of pharmacology.

Table 1 lists the problematic terms, their suggested replacements, and the rationale. The term antidepressants should be replaced by norepinephrine/serotonin (NE/5-HT) enhancers, describing the overarching mechanism of action of these drugs (Di Giovanni et al. 2016; Marazziti 2017; Cheffer et al. 2018). Additionally, the term NE/5-HT enhancer has a more positive connotation than antidepressant and avoids use of the term depression which has a strong negative bias. It will be much easier for a physician to prescribe a NE/5-HT enhancer than an antidepressant for obsessive-compulsive disorder or anxiety disorder.

**Table 1:** Problematic terms for psychoactive drugs and their replacements

Problematic term	Suggested replacement	Suggested abbreviated replacement	Rationale for using replacement	References
Antidepressant (drug)	Norepinephrine/serotonin enhancer	NE/5-HT enhancer	The indications for these drugs have expanded tremendously over the past years. The striking mismatch between drug class designation and clinical use renders literature research and patient communication complicated and prone to errors. The term antidepressant may stigmatize the patient.	Kenakin 2008; Skapinakis et al. 2016; Locher et al. 2017; Seifert 2018; Vries et al. 2018; Amerio et al. 2019
Antiepileptic (drug)	Neuronal inhibitor with pleiotropic effects	NIPE	See antidepressants. The term antiepileptic may stigmatize the patient.	Kenakin 2008; Seifert 2018
Antipsychotic (drug)	Antagonist at multiple G protein–coupled receptors	mGPCR antagonist	See antidepressants. The term antipsychotic may stigmatize the patient.	Kenakin 2008; Seifert 2018
Typical antipsychotic (drug)	Antagonist at multiple G protein–coupled receptors with preference for the dopamine D_2_-receptor	D_2_R-mGPCR antagonist	The original reason for using the term is not valid anymore, i.e., atypical antipsychotics cause EPSs as well. The new term emphasizes high affinity of the drugs for the D_2_R.	Fischer-Barnicol et al. 2008; Kenakin 2008; Divac et al. 2014; Kusumi et al. 2015; Seifert 2018; Hirano 2018
Atypical antipsychotic (drug)	Antagonist at multiple G protein–coupled receptors with pleiotropic effects	p-mGPCR antagonists	The original reason for using the term is not valid anymore, i.e., atypical antipsychotics cause EPSs as well. The new term emphasizes pleiotropic pharmacological profiles, therapeutic uses, and adverse effects.	Fischer-Barnicol et al. 2008; Kenakin 2008; Divac et al. 2014; Kusumi et al. 2015; Sykes et al. 2017; Seifert 2018
Mood stabilizer	Neuronal inhibitor with pleiotropic effects	mGPCR antagonist	See antidepressants	Kenakin 2008; Seifert 2018

The term neuroleptic drug is used synonymously to antipsychotic drug. The terms first-generation and conventional antipsychotics are used synonymously to typical antipsychotic (neuroleptic) drugs. The term second-generation antipsychotic (neuroleptic) is used synonymously to atypical antipsychotic (neuroleptic) drug. However, the term non-conventional antipsychotic (neuroleptic) drug is not used in the literature

The antiepileptics (anticonvulsants) comprise many heterogeneous groups of drugs with different mechanisms of action including sodium and calcium channel blockade and inhibition of glutamatergic mechanisms (Kobayashi et al. 2019). Thus, as a new umbrella term, we propose to use the term neuronal inhibitors with pleiotropic effects because the overall mechanism of action of these drugs is to reduce neuronal excitability. The term pleiotropic alludes to the fact that neuronal inhibition can be exploited in numerous indications.

The term mood stabilizer should also be abandoned because it is not clearly defined. Certain antiepileptics have mood-stabilizing properties as has the alkali ion lithium. Therefore, lithium could be placed into the group of NIPEs.

The term antipsychotics should be replaced by antagonists at multiple GPCRs (mGPCR antagonists) because this is the molecular mechanism underlying all antipsychotics (Gray and Roth 2007; Kahn et al. 2015; Kondej et al. 2018). The term mGPCR antagonists is neutral with respect to clinical use and readily allows for expansion of clinical uses. As another advantage, the efficacy of mGPCR antagonists in so many different psychiatric diseases indicates that, although phenotypically quite different, they may have a common pathophysiological basis. As pointed out above, the terms first generation/typical/conventional antipsychotics/neuroleptics as well as second-generation/atypical and unconventional antipsychotics are arbitrary and, hence, dispensable.

## Proposed solution to the problem—Step 2: Set up a mechanism-based nomenclature

In the preceding section, a number of broad new mechanism-based terms for psychotropic drugs were proposed. These terms describe systems that can be further divided into various drug classes which are represented by various drugs possessing various indications. Table 2 presents this system with 4 hierarchy levels (system ➔ drug class ➔ individual drug ➔ indication). The advantage of the system is that indications are at the lowest hierarchy level and can be readily adjusted for any given drug without affecting higher hierarchy level terms.

**Table 2:** A system-based nomenclature of psychoactive drugs

System	Drug class	Representative drugs	Representative indications	References
NE/5-HT enhancer	α_2_AR antagonists	Mirtazapine	Depression	Saltiel et al. 2015; Di Giovanni et al. 2016
			Panic disorder	Zulfarina et al. 2019
	NSMRIs	Amitriptyline	depression	Di Giovanni et al. 2016; Taciak et al. 2018
			Postherpetic neuralgia	Mallick-Searle et al. 2016
		Clomipramine	Depression	Di Giovanni et al. 2016; Taciak et al. 2018
			OCD	Del Casale et al. 2019
	SSRIs	Citalopram	Depression	Locher et al. 2017; Taciak et al. 2018
			Panic disorder	Andrisano et al. 2013
		Sertraline	Depression	Locher et al. 2017; Taciak et al. 2018
			PTSD	Graham et al. 2018
	SSNRIs	Venlafaxine	Depression	Di Giovanni et al. 2016; Locher et al. 2017; Taciak et al. 2018
			Generalized anxiety disorder	Slee et al. 2019
NIPEs	Calcium channel blockers	Pregabalin	Epilepsy	Kaufman 2011; Kobayashi et al. 2019
			Neuropathic pain	Hirakata et al. 2018
			Restless legs syndrome	Salminen and Winkelmann 2018
			Generalized anxiety disorder	Slee et al. 2019
	Sodium channel blockers	Carbamazepine	Epilepsy	Kaufman 2011; Kobayashi et al. 2019
			Bipolar disorder	Baldessarini et al. 2019
		Lamotrigine	Epilepsy	Kaufman 2011; Kobayashi et al. 2019
			BPD	Stone 2019
			Bipolar disorder	Baldessarini et al. 2019
		Valproic acid	Epilepsy	Kaufman 2011; Kobayashi et al. 2019
			Bipolar disorder	Baldessarini et al. 2019
			Migraine	Parikh and Silberstein 2019
	Inhibitors of glutamate release	Topiramate	Epilepsy	Kobayashi et al. 2019
			Essential tremor	Chang et al. 2015
			Migraine	Parikh and Silberstein 2019
	Alkali metal ions	Lithium	BPD	Stone 2019
			Bipolar disorder	Baldessarini et al. 2019
			Depression	Kleeblatt et al. 2017
mGPCR antagonists	D_2_R-mGPCR antagonists	Haloperidol	Schizophrenia	Kahn et al. 2015; Siskind et al. 2016
			Tic disorders	Pringsheim et al. 2019
	p-mGPCR antagonists	Clozapine Melperone Olanzapine Opipramol Pipamperone Quetiapine Risperidone	Schizophrenia	Bai et al. 2017; Vermeulen et al. 2019; Siskind et al. 2016
			Irritability in autism	Fallah et al. 2019
			Generalized anxiety disorder	Slee et al. 2019
			Bipolar disorder	Baldessarini et al. 2019
			Depression	Kleeblatt et al. 2017; Ceskova and Silhan 2018

*PTSD* post-traumatic stress disorder; *BPD* borderline personality disorder; *OCD* obsessive-compulsive disorder

The system of NE/5-NT enhancers is divided into the drug classes of α_2_AR antagonists, NSMRIs, SSRIs, SSNRIs, and MAO inhibitors. Additional drug classes can be readily added. Each drug class is then populated with various drugs to which specific indications are assigned.

The system of NIPEs is divided into various drug classes according to mechanism of action. Lithium is integrated into this system as well. However, since the mechanism of action of lithium is not yet known, a mechanistic class assignment is problematic. Rather, a chemistry-based term (alkali ion) is used. This neutral term could be replaced by a mechanism-based term at a later stage.

With respect to the mGPCR antagonists, one can define two broad classes of drugs. On one hand, we have drugs, haloperidol being the prototype, with affinity to multiple GPCRs and a high affinity to the D_2_R. These drugs can be designated as D_2_R-mGPCR antagonists. These drugs are characterized by a high risk of EPSs (Sykes et al. 2017). Many mGPCR antagonists are characterized by drug-specific pharmacological profiles, and it is rather difficult to assign a specific affinity for a given receptor to a given neuropsychiatric clinical effect. In addition, these drugs may or may not have the potential for EPSs. For example, clozapine has a low EPS risk, but other drugs such as risperidone have a higher EPS risk (Azorin et al. 2001). Moreover, the clinical uses of the drugs vary broadly (see Table 2). Accordingly, all drugs that are not characterized by a high D_2_R affinity relative to other GPCRs could be classified as pleiotropic mGPCR antagonists (p-mGPCR antagonists), alluding to the fact that these drugs have pleiotropic receptor profiles, pleiotropic therapeutic effects, and pleiotropic adverse reactions. This classification avoids making the incorrect distinction between drugs with high EPS risk and no EPS risk.

A major mechanistic implication of the change from antipsychotics to mGPCR antagonists is the fact that, in the new terminology, the words “GPCR” and “antagonist” are present. This is clinically relevant for three reasons. First, drugs acting via GPCRs show rapid clinical effects as compared with NE/5-HT enhancers. Second, GPCR antagonists, unlike GPCR agonists, do not exhibit desensitization and loss of efficacy during long-term therapy. Accordingly, in general, the drug dose does not have to be increased during long-term therapy. Third, GPCR antagonists do NOT cause addiction. This is a very important piece of information for patients who often fear that all psychoactive drugs cause addiction and tolerance. Last but not least, the terminology is consistent with other pharmacological fields; i.e., we have the terms H_1_R antagonists, β_1_AR antagonists, and M_x_R antagonists, to name few examples. The fact that in case of antipsychotics not a single GPCR is mentioned but rather GPCRs in general reflects the fact that these drugs bind to multiple receptors and have diverse therapeutic and adverse effects. Thus, the new nomenclature will, after some adjustment, render drug therapy and literature searches easier and logical and increase patient adherence.

## Where do we stand now?

It is beginning to emerge that, in many fields of medicine, a system- and mechanism-based approach is much more useful than the traditional organ- and disease-specific approach (Saltiel et al. 2015; Ramos and Bentires-Alj 2015; Gebicke-Haerter 2016; Walter et al. 2016; Bennett 2017; Zhou et al. 2018; Willsey et al. 2018). This strategy can and should be readily expanded to pharmacology as a whole and psychopharmacology in particular. The problem is that several generations of physicians, scientists, and pharmacists have learned the traditional nomenclature and are used to work with the system despite its apparent weaknesses and problems. Thus, it cannot be expected that the present proposal is enthusiastically embraced by all members of the medical and scientific community immediately. It is a process.

In Germany, the medical educational system has recently agreed on a mechanism-based drug nomenclature. This nomenclature has been published by the Institut für Medizinische and Pharmazeutische Prüfungsfragen (IMPP, Institute for Medical and Pharmaceutical Exam Questions) in November 2019 (IMPP-GK2, 5th edition, effective spring 2022): https://www.impp.de/pruefungen/allgemein/gegenstandskataloge.html. To integrate this nomenclature into pharmacology teaching of medical students, one of the authors of this article has written a textbook in English language (Seifert 2019) and German language (Seifert 2020) using the new nomenclature.

## General discussion

It must be emphasized that this article reflects the position of the authors but not international learned societies such as the International Union of Pharmacologists (IUPHAR). A logical next step will be to discuss this proposal at the IUPHAR level. The discussion of the topic will also have to involve psychiatrists. A better molecular characterization of psychiatric diseases and improved understanding of their pathophysiology will improve the matching of diseases to appropriate drugs (Kahn et al. 2015; Hirschtritt and Insel 2018; Kim and Park 2019).

It is also acknowledged that the proposed nomenclature does not only solve some problems but also creates problems. For example, one may argue that placing lithium with a still unknown mechanism of action (Won and Kim 2017) into the group of NIPEs leads to a substantial loss in granularity. Along the same line, some mGPCR antagonists (such as diphenhydramine) are predominantly used for the treatment of non-psychiatric diseases (Palmer et al. 2020; Fein et al. 2019), and how many GPCRs does a drug have to antagonize to be classified as mGPCR antagonist? Does, e.g., propranolol fulfill the criteria of a mGPCR antagonist (Cernecka et al. 2014)? Last but not least, how can we integrate pluridimensional efficacy, biased signaling, and allosteric GPCR modulators (Hauser et al. 2017; Seyedabadi et al. 2019; Kenakin 2019) into the classification without rendering it too complicated for the physician? We hope that, with this article, we initiate a discussion at an international level that contributes to improving drug classification of, and communication about, psychotropic drugs and ultimately improves drug safety.

## Electronic supplementary material

ESM 1 (DOCX 12 kb)

ESM 2 (XLSX 32 kb)

ESM 3 (XLSX 34 kb)
