# Supplementary material for: A simple mechanistic terminology of psychoactive drugs: a proposal
Source: Naunyn Schmiedebergs Arch Pharmacol. 2020 Jun 13;393(8):1331–9. doi: 10.1007/s00210-020-01918-x (PMC7351828; doi:10.1007/s00210-020-01918-x)
Supplement: Supplementary file 1 — (DOCX 12 kb) [file 210_2020_1918_MOESM1_ESM.docx]

**Supplementary Table 1: PubMed search details**

| **Figure** | **Panel** | **search term** | **Applied filters** |
| --- | --- | --- | --- |
| 1 | A | (anticonvulsant[Title/Abstract]) OR  (anticonvulsants[Title/Abstract]) | Journal Article,  MEDLINE |
|  | B | (antiepileptic[Title/Abstract]) OR  (antiepileptics[Title/Abstract]) |  |
|  | C | ("mood stabilizer"[Title/Abstract]) OR  ("mood stabilizers"[Title/Abstract]) OR  ("mood-stabilizing drug"[Title/Abstract]) OR  ("mood-stabilizing drugs"[Title/Abstract]) |  |
|  | D | (antidepressant[Title/Abstract]) OR  (antidepressants[Title/Abstract]) OR  ("antidepressive drug"[Title/Abstract]) OR  ("antidepressive drugs"[Title/Abstract]) |  |
|  | E | (antipsychotic[Title/Abstract]) OR  (antipsychotics[Title/Abstract]) |  |
|  | F | (neuroleptic[Title/Abstract]) OR  (neuroleptics[Title/Abstract]) |  |
| 2 | A | ("first-generation antipsychotic"[Title/Abstract]) OR ("first-generation antipsychotics"[Title/Abstract]) OR ("first-generation neuroleptic"[Title/Abstract]) OR  ("first-generation neuroleptics"[Title/Abstract]) |  |
|  | B | ("second-generation antipsychotic"[Title/Abstract]) OR  ("second-generation antipsychotics"[Title/Abstract]) OR  ("second-generation neuroleptic"[Title/Abstract]) OR  ("second-generation neuroleptics"[Title/Abstract]) |  |
|  | C | ("typical antipsychotic"[Title/Abstract]) OR  ("typical antipsychotics"[Title/Abstract]) OR  ("typical neuroleptic"[Title/Abstract]) OR  ("typical neuroleptics"[Title/Abstract]) |  |
|  | D | ("atypical antipsychotic"[Title/Abstract]) OR  ("atypical antipsychotics"[Title/Abstract]) OR  ("atypical neuroleptic"[Title/Abstract]) OR  ("atypical neuroleptics"[Title/Abstract]) |  |
|  | E | ("conventional antipsychotic"[Title/Abstract]) OR ("conventional antipsychotics"[Title/Abstract]) OR ("conventional neuroleptic"[Title/Abstract]) OR ("conventional neuroleptics"[Title/Abstract]) |  |
|  | F | (("*drug X*"[Title/Abstract]) AND ("extrapyramidal side effect"[Title/Abstract])) OR  (("*drug X* "[Title/Abstract]) AND ("extrapyramidal side effects"[Title/Abstract])) OR  (("*drug X* "[Title/Abstract]) AND ("extrapyramidal symptom"[Title/Abstract])) OR  (("*drug X* "[Title/Abstract]) AND ("extrapyramidal symptoms"[Title/Abstract])) |  |
